# Encoder–Decoder Architecture for 3D Seismic Inversion

**DOI:** 10.3390/s23010061

**Published:** 2022-12-21

**Authors:** Maayan Gelboim, Amir Adler, Yen Sun, Mauricio Araya-Polo

**Affiliations:** 1 Electrical Engineering Department, Braude College of Engineering, Karmiel 2161002, Israel; 2 TotalEnergies, EP R&T, Houston, TX 77002, USA

**Keywords:** 3D reconstruction, seismic inversion, seismic velocity, inverse problems, deep learning, transfer learning, encoder–decoder

## Abstract

Inverting seismic data to build 3D geological structures is a challenging task due to the overwhelming amount of acquired seismic data, and the very-high computational load due to iterative numerical solutions of the wave equation, as required by industry-standard tools such as Full Waveform Inversion (FWI). For example, in an area with surface dimensions of 4.5 km × 4.5 km, hundreds of seismic shot-gather cubes are required for 3D model reconstruction, leading to Terabytes of recorded data. This paper presents a deep learning solution for the reconstruction of realistic 3D models in the presence of field noise recorded in seismic surveys. We implement and analyze a convolutional encoder–decoder architecture that efficiently processes the entire collection of hundreds of seismic shot-gather cubes. The proposed solution demonstrates that realistic 3D models can be reconstructed with a structural similarity index measure (SSIM) of 0.9143 (out of 1.0) in the presence of field noise at 10 dB signal-to-noise ratio.

## 1. Introduction

A key step into understanding the subsurface by remote sensing, is the acquisition of seismic data, which consists of the recorded response of the subsurface when mechanical perturbations are introduced. After data has been collected, several disciplines of geoscience are involved towards the common objective of producing a reliable subsurface model(s). Earth models can be used for many purposes, such as: seismology studies, hydrocarbon exploration and CO_2_ sequestration. When used for the later purpose, models are critical inputs to drilling decisions. The problem at hand is daunting, involving too many variables and huge datasets. An example of great societal importance is injecting CO_2_ from industrial processes into specially reconditioned reservoirs. To that end, having high quality subsurface models is crucial. The solution of 3D seismic inverse problems using deep learning (DL) [1,2] is an emerging field of research, motivated by state-of-the-art results obtained by DL for the 2D case [3,4]. In particular, DL has been applied for velocity inversion [5,6,7,8,9,10,11,12,13], impedance inversion [14,15,16], reflectivity inversion [17,18,19] and low-frequency extrapolation [20,21,22,23,24]. In this study, we address the problem of 3D velocity inversion in large scale areas with hundreds of seismic shot-gather cubes, as required in realistic seismic surveys. The proposed solution is demonstrated in an area with surface dimensions of 4.5 km × 4.5 km, which requires over 500 seismic shot-gather cubes for 3D model reconstruction. Nevertheless, our solution is scalable to larger area dimensions and higher numbers of shot-gather cubes, facilitated by the utilization of a dimensionality reduction approach. The contributions of this paper are threefold: (1) A convolutional encoder–decoder network is proposed with an efficient input data dimensionality reduction and time boosting of all seismic traces, to reconstruct complex 3D models at an average inference time of 0.165 s (on one NVIDIA A100 GPU), which is a fraction of the time required by any iterative global optimization solver. (2) The proposed approach is demonstrated to provide inherent robustness against noise in the recorded seismic data. (3) The proposed approach is successfully evaluated with realistic 3D geological models and field noise.

## 2. Problem Formulation

Direct reconstruction of models of solid earth is not possible, this renders the following forward model:

(1) d=F(m)+ϵ,

only practical when synthetic seismic data (d) is to be generated from a forward operator F acting on a artificial model m, under ambient noise ϵ. F approximates the behavior of seismic waves propagating through the mechanical medium (m), and it is represented by the following expression:

(2) $$\frac{\partial^{2}u}{\partial t^{2}}-V_{P}^{2}\nabla \left(\nabla \cdot u\right)+V_{S}^{2}\nabla \times \left(\nabla \times u\right)=f,$$

where u=u(x,y,z,t) is the seismic wave displacement, $V_{P}$ is P-wave velocity (compression/rarefaction), $V_{S}$ is S-wave velocity (shear stress), and f is the source function. While the elastic (in the presence of P-wave and S-wave attenuation the viscoelastic wave equation is utilized instead of the elastic equation) wave equation describes faithfully seismic waves propagation, it is often preferred (as in this work) to approximate it by the acoustic wave equation [25], which assumes only P-waves and requires less computational resources and parameters, as compared to solving the elastic equation. The acoustic wave equation for a medium without density variations is given by:

(3) $$\frac{\partial^{2}u}{\partial t^{2}}-V^{2}\nabla^{2}u=f,$$

where u is the wave displacement, V is the P-wave velocity model and f is the perturbation source (i.e., *shot*) function.

Since the direct formulation is not tractable, it is common to use the inverse approach. Seismic velocity inversion computes a complete 3D velocity model ($\hat{m}$) of a certain target area, from recorded seismic data $d_{r}$, and it can be summarized as:

(4) $$\hat{m}=F^{-1}\left(d_{r}\right),$$

where $F^{-1}$ is the inversion operator. Seismic inversion problems [26] are ill-posed, e.g., the solution is non-unique and unstable in the sense that small noise variations may alter the solution significantly. The DL formulation for solving inverse problems is detailed in the next section.

## 3. The Deep Learning Approach

### 3.1. Encoder–Decoder Architecture

Deep Learning (DL) is a powerful class of data-driven machine learning algorithms, built using Deep Neural Networks (DNNs), which are formed by a hierarchical composition of non-linear functions (layers). The main reason for the success of DL is the ability to train very high capacity networks using very large datasets, often leading to good *generalization* capabilities in numerous problem domains. Generalization is defined as the ability of an algorithm to perform well on unseen examples. In statistical learning terms an algorithm A:X→Y is learned using a training dataset $S=\{\left(x_{1},y_{1}\right),...,\left(x_{N},y_{N}\right)\}$ of size *N*, where $x_{i}\in X$ is a data sample (in this work, a seismic shot-gather) and $y_{i}\in Y$ is the corresponding label (in this work, a 3D velocity model). Let P(X,Y) be the true distribution of the data, then the expected risk is defined by: $R\left(A\right)=E_{x,y∼P(X,Y)}\left[L\left(A\left(x\right),y\right)\right],$ where L is a loss function that measures the misfit between the algorithm output and the data label. The goal of DL is to find an algorithm A within a given capacity (i.e., function space) that minimizes the expected risk; however, the expected risk cannot be computed since the true distribution is unavailable. Therefore, the empirical risk is minimized instead: $R_{E}\left(A\right)=\frac{1}{N}\sum_{i=1}^{N}L\left(A\left(x_{i}\right),y_{i}\right),$ which approximates the statistical expectation with an empirical mean computed using the training dataset.

**Figure 1:**
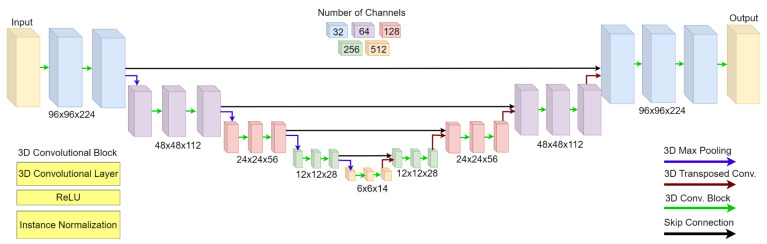
The proposed 3D Encode-Decoder architecture, based on the U-Net architecture. The skip connections perform a replication of the encoder feature maps, which are further concatenated with the corresponding decoder feature maps. Additional details are provided in Table 1.

In this work we implemented and trained a 3D convolutional encoder–decoder, inspired by the 2D U-Net architecture [27], to learn the mapping from seismic data space to 3D models space (i.e., inversion). The complete network architecture is depicted in Figure 1, and the details of each block are provided in Table 1, with a total of 99M parameters.

### 3.2. Computational Considerations

The main challenge in training such a deep convolutional neural network (DCNN) for real-life inversion tasks lies in the demanding GPU RAM size and external storage access requirements due to the large number of input channels and large size of each input channel: each sample in our training data was composed of $N_{x}\times N_{y}=529$ seismic data cubes (i.e., DCNN input channels), where $N_{x},N_{y}$ are the total numbers of shots in the lateral and longitudinal axes, respectively. Therefore, a total storage size of 42GB per sample (after decimation to dimensions 96×96×224). A modest training dataset of 800 samples occupies ≈4TB storage size, which requires very high-speed storage access to facilitate DCNN training in reasonable duration. Thus, the problem belongs to a High-performance Computing class [28]. To overcome these challenging requirements, we propose a simple yet highly-effective dimensionality reduction scheme: let $d(S_{x},S_{y},R_{x},R_{y},t)$ denote the 5D tensor that represents a single data sample, i.e., the collection of seismic data cubes (shot-gathers), where $S_{x},S_{y}$ are the indices of the shot position, $R_{x},R_{y}$ are the indices of the receiver position, and *t* is time. We define the time-boosted and dimensionality-reduced data cube $\bar{d}$ by spatial averaging along the shots dimensions:

(5) $$\bar{d}\left(R_{x},R_{y},t\right)=\frac{b(t)}{N_{x}\times N_{y}}\sum_{S_{x}=1}^{N_{x}}\sum_{S_{y}=1}^{N_{y}}d(S_{x},S_{y},R_{x},R_{y},t),$$

where b(t) is a monotonically-increasing time-boosting function that compensates the attenuation of wave reflections from the lowest geological layers, by amplifying late-arrival time samples. Therefore, $\bar{d}$ forms a single 3D input channel, thus significantly mitigating the memory and computational requirements for training and inference of the proposed DCNN.

In the next section, we describe the performance of the proposed architecture for noiseless seismic data, as well as data contaminated by synthetic and field noise.

## 4. Performance Evaluation

### 4.1. Data Preparation

**Figure 2:**
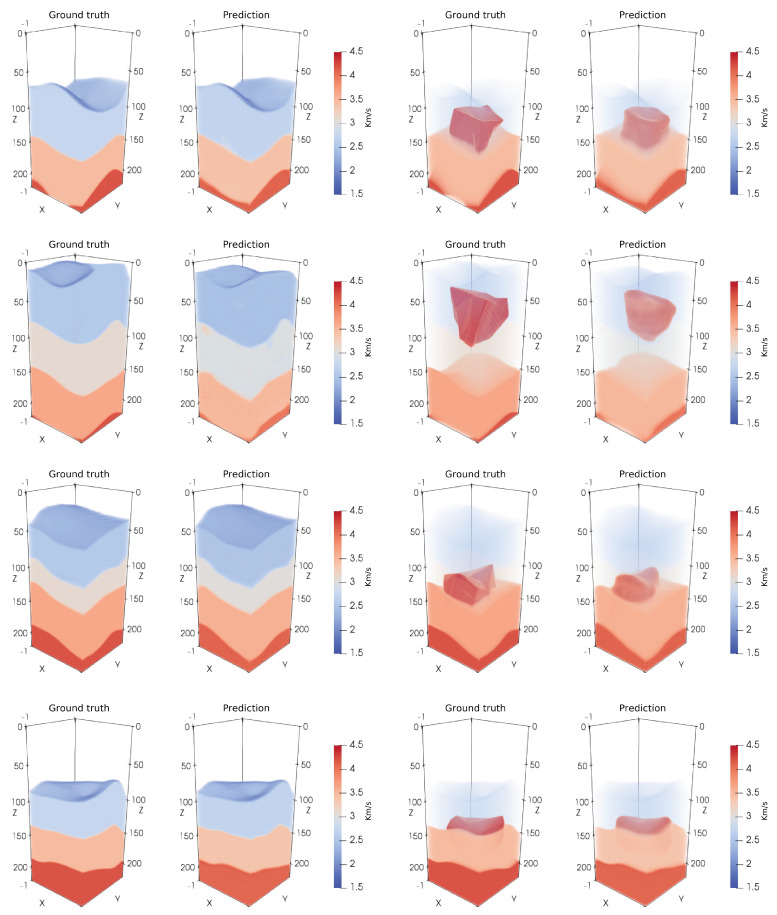
3D inversion results of four velocity models with salt geometry, from the held-out testing set. Each row presents one model: the two left columns display the ground-truth and the corresponding inverted model. In the two right columns, the shallow layers (slow velocity) are removed to enable the visualization of the embedded salt bodies.

We created 800 3D velocity models using the Gempy (https://www.gempy.org/, accessed on 1 October 2020) tool that creates 3D geologically-feasible models with realistic combinations of features. The selection of Gempy as subsurface modeler is not arbitrary, and obeys to the intention of solving a more realistic problem than just flat layer-cake models. A subset of 300 models were augmented with random 3D geometries that resemble salt structures, as illustrated in Figure 2. The physical dimensions of each model were 4.5 km × 4.5 km × 4.0 km (lateral × longitudinal × depth), represented by a 3D tensor of dimensions 300×300×800 grid points, which was down-sampled for DCNN training to dimensions of 96×96×224. To generate the synthetic seismic data, through forward modeling, we use an acoustic isotropic wave equation propagator with a 15 Hz peak frequency Ricker wavelet as a source. Shots and receivers are evenly spaced on the top surface of the 3D model (200 m between shots and 25 m between receivers). To avoid reflections from the boundaries and free surface multiples, convolutional perfectly matched layer (CPML) [29] boundaries are imposed all around the model. Each generated seismic data cube was computed on a grid of dimensions 180×180×500 (lateral × longitudinal × time) points, which was down-sampled for DCNN training to dimensions of 96×96×224.

**Figure 3:**
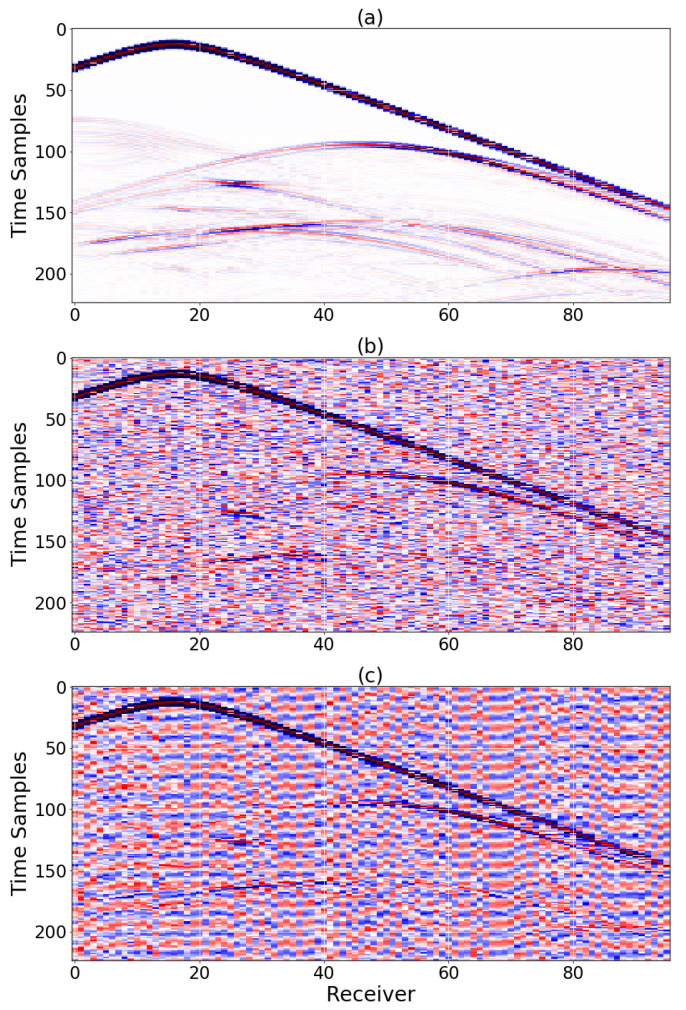
A 2D slice of a 3D shot-gather: (**a**) noiseless. (**b**) distorted by additive white Gaussian noise (SNR = 10 dB). (**c**) distorted by additive colored field noise (SNR = 10 dB).

The 800 3D models were split to disjoint training and testing sets by a 90%/10% ratio, respectively. The proportions of models without and with salt structures were identical in the training and testing sets, namely, 450 models without salt and 270 with salt in the training set, and 50 models without salt and 30 with salt in the testing set. The proposed DCNN was implemented in PyTorch and trained using the NVIDIA A100 Tensor Core GPU card (40 GB RAM). Training was performed using the ADAM optimizer with early stopping regularization, by minimizing the Mean Absolute Error (MAE) loss function, defined by:

(6) $$\mathrm{MAE}\left(X,Y\right)=\frac{1}{N}\sum_{i=1}^{N}\left|x_{i}-y_{i}\right|,$$

where $x_{i},y_{i}$ are the grid point entries of the ground truth 3D model X and inverted 3D model Y, respectively (each model with *N* grid points). Training was initially performed for the noiseless data case, and subsequently using transfer learning, for the six noisy cases. For each type of noise training started from the learned weights of the noiseless case (we found this approach to provide a significant advantage as compared to training from random weights, for each noisy data case). This process resulted in seven different trained DCNNs: noiseless data, data contaminated with white Gaussian noise at signal-to-noise (SNR) levels of 20 dB, 10 dB and 0 dB, and data contaminated with noise extracted from field data (from recordings of an onshore field) at SNR levels of 20 dB, 10 dB and 0 dB. Figure 2 presents 3D inversion results from noiseless data of four velocity models with salt geometry, from the held-out testing set. The results clearly indicate high-quality 3D reconstruction of the geological layers and salt bodies. Examples of the clean and noisy data are provided in Figure 3, demonstrating the highly correlated patterns in space- and time-domains of the field noise.

### 4.2. Evaluation Metrics

**Figure 4:**
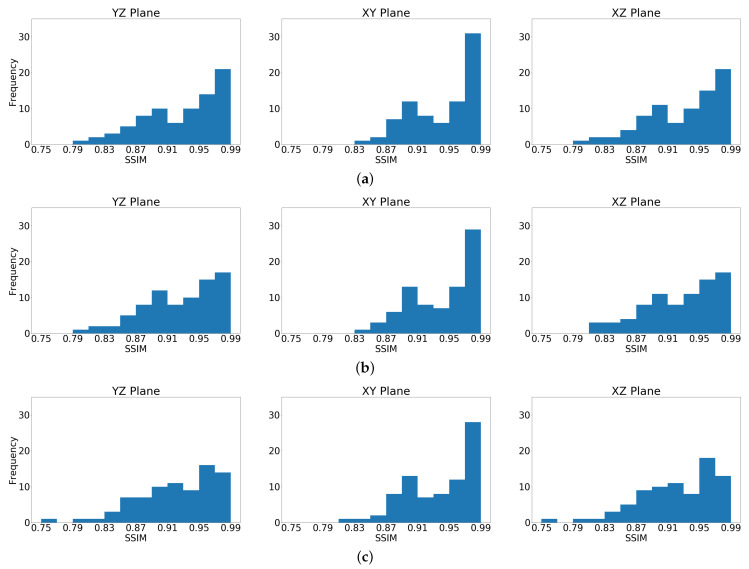
SSIM values histograms of all testing set samples, computed separately along the three 2D planes: (**a**) noiseless data. (**b**) data contaminated by **white** noise at SNR = 20 dB; and (**c**) data contaminated by **field** noise at SNR = 20 dB. The histograms clearly indicate that most SSIM values are distributed between 0.80 and 0.99. The average 3D SSIM values are 0.9335 for the noiseless case, 0.9316 with **white** noise (SNR = 20 dB) and 0.9271 with **field** noise (SNR = 20 dB).

**Figure 5:**
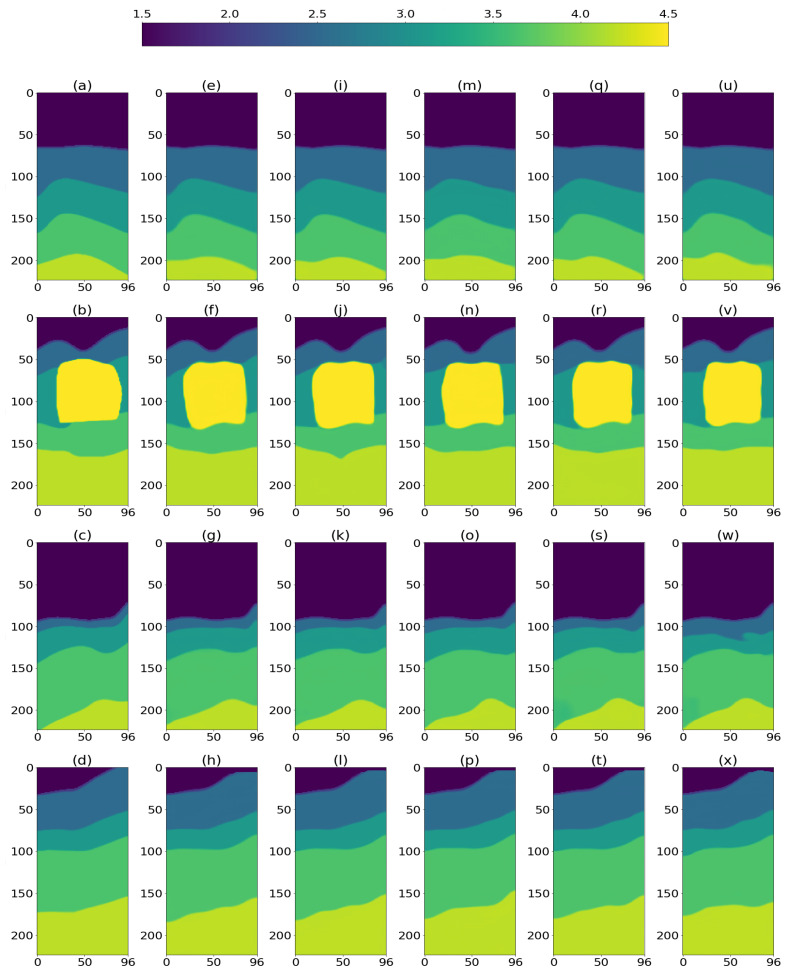
2D cross-sections of reconstructed 3D models from unseen data: (**a**–**d**) ground truth. (**e**–**h**) reconstruction from noiseless data. (**i**–**l**) reconstruction from noisy data: white noise, SNR = 20 dB; and (**m**–**p**) white noise, SNR = 10 dB. (**q**–**t**) reconstruction from noisy data: field noise, SNR = 20 dB; and (**u**–**x**) field noise, SNR = 10 dB.

SSIM [30] results were computed per 3D model by first averaging SSIM values along the three 2D planes: 96 along the XZ plane, 96 along the YZ plane, and 224 along the XY plane. Finally, the three results were averaged to obtain the single SSIM(3D) result. The distribution of SSIM values, as computed along the three 2D planes (XY, YZ, XZ), for the entire testing set is presented in Figure 4, demonstrating accurate reconstruction along the three 2D planes, with slightly lower SSIM values for the vertical planes (YZ, XZ), which can be explained by the difficulty to reconstruct the deepest layers. Table 2, details SSIM and MAE results, averaged on the testing set, clearly demonstrating that the proposed DCNN is capable to reconstruct 3D velocity models from noiseless data (Figure 5e–h), as well as with additive white noise (Figure 5i–p) or field noise (Figure 5q–x). Importantly, results for seismic data contaminated by field noise, indicate close similarity to the ground truth models at a SNR of 20 dB, but the SSIM metric slightly deteriorated at SNR of 10 dB.

**Figure 6:**
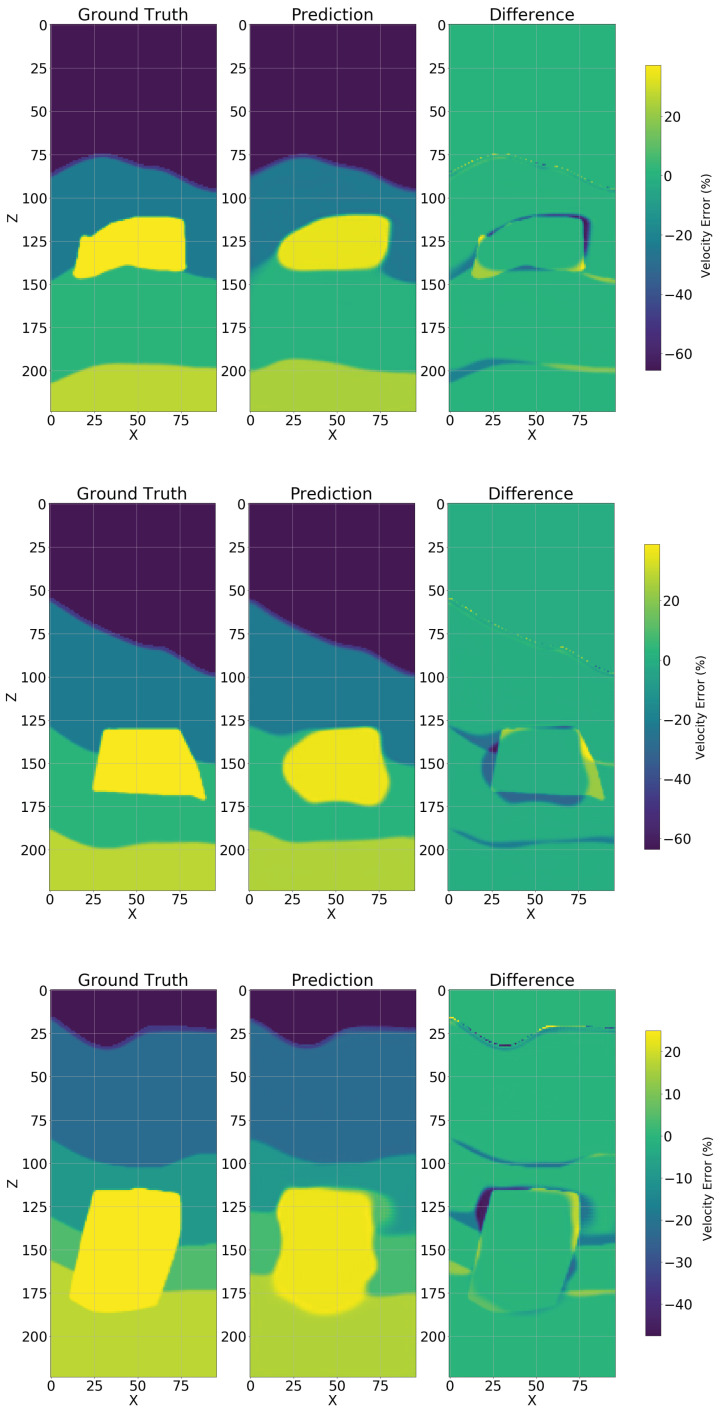
Three testing samples were selected and a 2D (central inline) cut is shown, a comparison between ground-truth and prediction is presented in terms of error. As expected, the greater error (right most bar, in percentage) occurs around the salt geometry and mostly over estimating the model velocity, nevertheless the background velocity is overall correct.

The results in the noiseless data case are surprising, indicating that the dimensionality-reduced data cube (5) contains sufficient information for practical reconstruction given the measured metric, achieving an average SSIM(3D) of 0.9003 by the DCNN. In addition, examples of the structure of the prediction error are presented in Figure 6. We next discuss the noisy data case.

### 4.3. Noisy Data Analysis

In the presence of additive white noise that is spatially (and temporally) independent and identically distributed (iid), the spatial averaging along the shots dimensions results in a reduction of the noise variance, as explained in the following analysis. Denoting by $n(R_{x},R_{y},t)$ the noise random variable resulting from the spatial averaging of noise samples, corresponding to receiver coordinates $\left(R_{x},R_{y}\right)$ and time *t*:

(7) $$\bar{n}\left(R_{x},R_{y},t\right)=\frac{1}{N_{x}\times N_{y}}\sum_{S_{x}=1}^{N_{x}}\sum_{S_{y}=1}^{N_{y}}n(S_{x},S_{y},R_{x},R_{y},t),$$

where $n(S_{x},S_{y},R_{x},R_{y},t)$ are iid random variables with zero mean and variance $\sigma_{n}^{2}$. By using the iid property, the variance of $\bar{n}\left(R_{x},R_{y},t\right)$ is independent of $R_{x},R_{y},t$ and given by $\sigma_{\bar{n}}^{2}\left(R_{x},R_{y},t\right)=\sigma_{\bar{n}}^{2}=\frac{1}{N_{x}\times N_{y}}\sigma_{n}^{2}$ (in our study $N_{x}\times N_{y}=529$). The time-boosting function b(t) is omitted from (7), since in the presence of additive noise, both the signal and noise components are multiplied by b(t), according to (5), thus the contribution of b(t) is cancelled in a SNR analysis. Therefore, the variance of the noise component in the dimensionality-reduced data cube is effectively reduced by $N_{x}\times N_{y}$, for iid white noise. However, the field noise is clearly not iid, therefore a smaller reduction in the noise variance is achieved.

## 5. Conclusions

Seismic inversion based on DL effectively reconstruct 3D subsurface models from synthetic seismic data and synthetic seismic data contaminated by either white or field noise. Once training is settled, the inference step is fast—a fraction of a second—to a point that allows many different experiments to be carried out with marginal cost. To allow feasible training time a dimensionality reduction technique is deployed. Also, the robustness to noise was demonstrated for practical SNR levels. The next steps for this approach are: training and testing with more complex and larger scale structures, and to estimate the sensitivity with respect to acquisition towards direct use of field data.

## Figures and Tables

**Table 1:** Proposed encoder–decoder architecture.

Block	Layer	Unit	Comments
Input	0	Seismic Cube	96×96×224 grid points
	1	Conv3D(32, (5×5×5), ReLU)	+ InstanceNormalization
Enc1	2	Conv3D(32, (5×5×5), ReLU)	+ InstanceNormalization
	3	MaxPool3D	+ Dropout(0.2)
Enc2	4–6	Enc1(64)	
Enc3	7–9	Enc1(128)	
Enc4	10–12	Enc1(256)	
Enc5	13–14	Enc1(512)	without MaxPool3D
	15	ConvTrans3D(256, (2×2×2), ReLU)	+ InstanceNormalization
Dec1	16	Conv3D(256, (5×5×5), ReLU)	+ InstanceNormalization
	17	Conv3D(256, (5×5×5), ReLU)	+ InstanceNormalization
Dec2	18–20	Dec1(128)	
Dec3	21–23	Dec1(64)	
Dec4	24–26	Dec1(32)	
	27	Conv3D(1, (1×1×1), ReLU)	final reconstruction layer
Output	28	Velocity Model	96×96×224 grid points

**Table 2:** 3D velocity model building quality comparison. All values are reported as: Mean(Std), MAE results are in [Km/s]. Results with salt geometry augmented models are included in this table.

Metric	Noiseless	White Noise	White Noise	White Noise	Field Noise	Field Noise	Field Noise
	Data	(SNR = 20 dB)	(SNR = 10 dB)	(SNR = 0 dB)	(SNR = 20 dB)	(SNR = 10 dB)	(SNR = 0 dB)
SSIM(3D)	0.9335 (0.0449)	0.9316 (0.0440)	0.9192 (0.0465)	0.8621 (0.0528)	0.9271 (0.0461)	0.9143 (0.0493)	0.8605 (0.0488)
SSIM(XZ)	0.9294 (0.0458)	0.9272 (0.0446)	0.9135 (0.0467)	0.8475 (0.0484)	0.9222 (0.0470)	0.9078 (0.0495)	0.8455 (0.0425)
SSIM(XY)	0.9433 (0.0388)	0.9417 (0.0394)	0.9329 (0.0409)	0.8960 (0.0425)	0.9385 (0.0407)	0.9296 (0.0427)	0.8953 (0.0401)
SSIM(YZ)	0.9280 (0.0471)	0.9257 (0.0460)	0.9113 (0.0484)	0.8426 (0.0497)	0.9207 (0.0481)	0.9054 (0.0515)	0.8407 (0.0438)
MAE(3D)	0.0380 (0.0349)	0.0394 (0.0343)	0.0490 (0.0428)	0.1214 (0.0716)	0.0426 (0.0375)	0.0550 (0.0510)	0.1206 (0.0667)

## Data Availability

Not applicable.
